# Seated Versus Standing Endoscopic Retrograde Cholangiopancreatography: Association with Operator Fatigue and Pain

**DOI:** 10.1055/a-2957-0590

**Published:** 2026-09-25

**Authors:** Kazuya Sumi, Jun Ushio, Minoru Daira, Hisaki Kato, Yuki Kawasaki, Tomoaki Kakazu, Yukiko Okada, Ryo Hashizume, Satoshi Ishijima, Takayoshi Ito, Noboru Yokoyama, Haruhiro Inoue

**Affiliations:** 1 Digestive Diseases Center 378609 Showa Medical University Koto Toyosu HospitalKoto-kuTokyoJapan; 2 Department of Rehabilitation 378609 Showa Medical University Koto Toyosu HospitalKotoJapan

**Keywords:** endoscopic retrograde cholangiopancreatography, musculoskeletal disorders, ergonomics, seated posture

## Abstract

**Background and Study Aims** Endoscopic retrograde cholangiopancreatography (ERCP) is a physically demanding procedure for operators that can provoke musculoskeletal symptoms. However, burden reduction and operating performance from a seated operating posture have not been established. Thus, we evaluated the association between seated operating posture and acute changes in operator fatigue and pain during ERCP.

**Patients and Methods** In this single-center observational study, 113 ERCP cases with paired pre- and postprocedure assessments were analyzed. Operator-reported global fatigue and pain were measured using numeric rating scales and expressed as pre- to postprocedure changes (ΔFatigue and ΔPain). Linear mixed-effects models were constructed to compare posture (seated vs. standing) and symptom changes, adjusting for procedure time and operator experience, with operator ID included as a random intercept. Feasibility and safety outcomes were also compared.

**Results** The seated posture was associated with significantly smaller increases in fatigue and pain than standing. In adjusted mixed-effects analyses, the seated posture remained independently associated with smaller increases in both ΔFatigue (estimate −1.650; *p* < 0.0001) and ΔPain (estimate −1.614; *p* < 0.0001), whereas longer procedure time was independently associated with greater ΔFatigue but not ΔPain. Procedural success rates, fluoroscopy-related parameters, and adverse events were comparable between groups. Operator-reported comfort was significantly higher in the seated group ( *p* < 0.0001).

**Conclusions** Seated ERCP was independently associated with reduced acute operator fatigue and pain, apparently without compromising procedural performance. These findings demonstrate that posture modification offers a simple and practical ergonomic approach to reducing operator burden during routine ERCP.

## Introduction

Musculoskeletal disorders (MSDs) in endoscopists are increasingly recognized as a significant occupational health concern. 1 2 3 4 5 6 7 Endoscopic procedures often demand prolonged static postures, repetitive movements, and sustained physical load, all of which contribute to acute fatigue and pain, thereby predisposing operators to chronic MSDs. As demand for complex therapeutic endoscopy increases, practical strategies that reduce operator burden while maintaining procedural quality and safety must be urgently identified.

Endoscopic retrograde cholangiopancreatography (ERCP) is a technically advanced endoscopic intervention that requires sustained visual–motor coordination, precise duodenoscope manipulation, and a relatively fixed body posture for extended periods. With expanding indications and increasing procedural complexity, ERCP procedures have become progressively longer and are among the most physically demanding for operators. 8 During ERCP, operators are required to wear protective lead aprons and coordinate simultaneous endoscopic and fluoroscopic visualization, while adjusting body position, as needed. These ergonomic requirements are often exacerbated by the confined environment of fluoroscopy rooms, making operator posture optimization a critically important issue in ERCP practice. 9 10 11

Most studies evaluating MSDs among endoscopists have relied on surveys assessing symptom prevalence over extended periods. 1 3 Although these studies have consistently demonstrated a high prevalence of musculoskeletal symptoms, few have assessed the ergonomic impact of specific procedural factors. Moreover, many proposed countermeasures focus on individual-level behavioral strategies, such as stretching exercises, ergonomic education, and scheduled breaks, which can be difficult to implement consistently in busy clinical environments. 1 3 12 13 14 Although these epidemiologic investigations have been insightful regarding the overall burden of MSDs, most have concentrated on the cumulative exposure over long time frames or across multiple endoscopic procedures. Consequently, the ergonomic impact of specific procedural factors at the level of individual ERCP sessions has not been thoroughly characterized.

Operator posture is a potentially modifiable ergonomic factor during ERCP. Although a standing posture has traditionally been employed for ERCP, prolonged static standing increases lower-extremity loading, venous pooling, and accumulation of general physical fatigue. 3 13 15 In contrast, performing ERCP in a seated posture has been proposed as a practical alternative, potentially reducing postural load while preserving fine motor control of an endoscope. 16 However, despite limited experience and feasibility reports, the comparative analysis of seated and standing postures during ERCP in real-world clinical conditions remains scarce, and concerns persist about the potential effects of posture on procedural performance.

In the context of increasing procedural volume, aging endoscopy operators, and the growing emphasis on occupational health and sustainability in medical practice, 1 4 clarifying the impact of seated ERCP on operator burden and technical outcomes is of substantial clinical relevance. Therefore, this study evaluated the association between a seated operating posture and favorable changes in operator burden and assessed the impact of a seated posture on procedural performance.

## Material and Methods

### Study Design and Participants

This retrospective observational study analyzed prospectively collected questionnaire data from ERCP procedures performed at Showa Medical University Koto-Toyosu Hospital between April and September 2025. Ergonomic questionnaires, including fatigue, pain, comfort, and newly developed musculoskeletal symptoms, were prospectively completed immediately after each ERCP. Patient characteristics and procedural variables were retrospectively retrieved from the medical and endoscopy records. The study was approved by the Institutional Review Board of Showa Medical University (approval number: 2025-0387) and conducted in accordance with the Declaration of Helsinki.

The study venue was a referral hospital that provided emergency care, where workflow and room availability varied with the clinical demands. Posture selection was determined by procedural circumstances and a room setup rather than a protocol-driven assignment, thus reflecting routine clinical practice. Questionnaires were collected only when the investigator was available to administer the assessment. Operators adjusted monitor and table heights at their comfort to maintain a visual relationship between the upper body and the monitor, similar to the conventional standing procedure ( **Figs. S1 and S2** ). All ERCP procedures were performed with the patient in the prone position. Seven endoscopists performed the ERCP procedures, including three experienced specialists and four trainees (6 men and 1 woman). Procedures performed by trainees were supervised by an experienced specialist. Only procedures completed entirely by the trainee without hands-on takeover by the supervising endoscopist were included in the analysis. All participating operators were actively engaged in ERCP practice during the study period and had no MSD requiring ongoing medical treatment, as indicated in their medical anamneses. All interventions were made either in the conventional standing position or in a seated posture. Five of the 7 participating operators performed ERCP in both seated and standing positions during the study period, while the remaining two performed standing ERCP only.

### Assessment and Outcomes

For each ERCP session, patient and procedural characteristics were recorded, including age, sex, indication categorized as benign or malignant disease, presence of surgically altered anatomy, and total procedure time. Fluoroscopy-related parameters, procedural success, and adverse events were documented to assess potential posture-related differences in technical performance.

Operator musculoskeletal burden was evaluated immediately before and after each ERCP procedure using a numeric rating scale (NRS; 0–10) to assess total fatigue and pain levels. 15 This scale was selected to capture the overall subjective symptom experience during a procedure.

The primary outcomes were pre- and postprocedure changes in total fatigue and pain NRS scores, calculated as the difference between the post- and preprocedure values.

Secondary outcomes included operator-reported comfort, scored on a 5-point Likert scale (1 = very uncomfortable to 5 = very comfortable), and the distribution of newly developed musculoskeletal discomfort by anatomical region (neck/shoulder, upper extremities, lower extremities, and lumbar region). New-onset symptoms were defined as those that were absent before a procedure that became apparent after it within the same anatomical region. Procedural metrics analyzing procedure time, technical success, and adverse events were also examined.

### Statistical Analysis

Continuous variables were expressed as mean (SD) if normally distributed and as median with interquartile range (IQR) if not. Categoric variables were expressed as counts and percentages. Continuous variables were compared using the Student *t* test or the Mann–Whitney *U* test, depending on whether the data were normally distributed. Categoric variables were compared using the chi-square or Fisher’s exact test, as appropriate.

For the primary analysis, linear mixed-effects models were constructed to analyze the relationship between procedural posture and changes in operator fatigue and pain. The dependent variables were ΔFatigue and ΔPain, each calculated as the postprocedure score minus the corresponding preprocedure score. Procedural posture (seated vs. standing), procedure time, and operator experience (specialist vs. trainee) were treated as fixed effects, and operator ID was included as a random intercept to account for within-operator clustering. A variance-components covariance structure was used for the random effect. Model parameters were analyzed using the restricted maximum likelihood method. Because ΔFatigue and ΔPain were treated as co-primary outcomes, statistical significance was assessed using a two-sided significance level of 0.025, and fixed-effect estimates are presented with 97.5% confidence intervals. To examine whether the association between procedural posture and operator burden differed according to procedure duration, additional mixed-effects models included an interaction term between procedural posture and procedure time. As a sensitivity analysis, the primary mixed-effects models were repeated after restricting the dataset to the five operators who performed ERCP in both seated and standing postures. No missing data were present in the analyzed dataset.

Secondary outcomes were compared between posture groups using the relevant statistical tests, as described earlier. Analyses of regional symptoms were considered exploratory.

All data were statistically analyzed using JMP Student Edition 19 (SAS Institute, Cary, NC, USA).

## Results

### Study Cohort and Procedural Characteristics

A total of 113 ERCP procedures with paired pre- and postprocedure assessments were covered by the final analysis. Baseline patient demographics and procedural characteristics were broadly comparable between seated and standing ERCP groups, including American Society of Anesthesiologists (ASA) physical status classification, the proportions of benign versus malignant indications, and the prevalence of surgically altered anatomy. The proportion of specialist and trainee operators differed significantly between the seated and standing groups ( Tables 1 and 2 ).

**Table 1:** Patient and procedural characteristics ( *n* = 113).

Baseline characteristics	Overall ( *n* = 113)
Age, years	74.8 (14.0)
Male sex, *n* (%)	65 (57.5%)
BMI, kg/m ^2^	21.5 (3.8)
Operator (Specialist), *n* (%)	62 (54.9%)
Procedural time, min	41.2 (25.7)
Radiation dose, mGy	213.2 (180.7)
Sedation (propofol), *n* (%)	113 (100%)
Posture (Seated), *n* (%)	54 (47.8%)

BMI: Body mass index.

**Table 2:** Baseline patient and procedural characteristics by operator posture.

Variable	Seated ( *n* = 54)	Standing ( *n* = 59)	*p* -Value
Age, years	73.5 (14.3)	76.0 (13.7)	0.342
Male sex, *n* (%)	30 (55.6%)	35 (59.3%)	0.707
BMI, kg/m ^2^	21.3 (3.9)	21.7 (3.7)	0.617
ASA-PS I–II, *n* (%)	34 (63.0%)	42 (71.2%)	0.424
ASA-PS III–IV, *n* (%)	20 (37.0%)	17 (28.8%)	
Anticoagulant use, *n* (%)	15 (27.8%)	17 (28.8%)	1.000
Diagnosis of malignancy, *n* (%)	14 (25.9%)	15 (25.4%)	1.000
Surgically altered anatomy, *n* (%)	7 (13.0%)	6 (10.2%)	0.771
Specialists, *n* (%)	37 (68.5%)	25 (42.4%)	0.005
Procedural time, min	41.4 (28.6)	41.1 (23.0)	0.956
Radiation dose, mGy	211.1 (188.8)	215.2 (174.5)	0.907
Propofol dose, mg	299.0 (178.0)	284.1 (147.6)	0.630

BMI, Body mass index; ASA-PS, American Society of Anesthesiologists physical status.

### Procedural Feasibility and Safety

Procedure time, fluoroscopy-related parameters, procedural success rates, and the incidence of adverse events had comparable values between groups, indicating that adoption of the seated posture did not adversely affect the technical performance in this cohort ( Table 3 ).

**Table 3:** ERCP procedural outcomes.

Variable	Seated ( *n* = 54)	Standing ( *n* = 59)	*p* -Value
Procedure success, *n* (%)	53 (98.1%)	59 (100%)	0.478
Presence of any adverse events, *n* (%)	1 (1.9%)	3 (5.1%)	0.620
PEP, *n* (%)	1 (1.9%)	2 (3.4%)	1.000
>3 × ULN amylase, *n* (%)	11 (20.3%)	9 (15.3%)	0.623
Post-ERCP abdominal pain, *n* (%)	3 (5.6%)	3 (5.1%)	1.000

ERCP, endoscopic retrograde cholangiopancreatography; PEP, post-ERCP pancreatitis; ULN, upper limit of normal.

### Changes in Operator Fatigue and Pain

Preprocedure fatigue and pain scores generally had similar values between the posture groups. Crude comparisons showed that seated ERCP was associated with significantly smaller increases in both fatigue and pain compared with standing ERCP ( Table 4 ). The median increase in fatigue was 0 (IQR −0.25 to 1) in the seated group compared with 1 (IQR 1 to 2) in the standing group. Similarly, the median increase in pain score was 0 (IQR −2 to 0) versus 1 (IQR 0 to 2), respectively.

**Table 4:** Fatigue and pain scores before and after ERCP.

Outcome	Seated ( *n* = 54)	Standing ( *n* = 59)	*p* -Value
Fatigue (pre-procedure), median [IQR]	3 (2–4.25)	3 (1–5)	0.657
Pain (pre-procedure), median [IQR]	2 (1–3)	1 (0–2)	0.580
Fatigue (post-procedure), median [IQR]	3 (2–5)	5 (3–7)	0.001
Pain (post-procedure), median [IQR]	1 (0–2)	2 (1–4)	<0.0001
ΔFatigue, median [IQR]	0 (−0.25 to 1)	1 (1–2)	<0.0001
ΔPain, median [IQR]	0 (−2 to 0)	1 (0–2)	<0.0001

ERCP, endoscopic retrograde cholangiopancreatography.

### Mixed-effects Analysis

The primary multivariate analysis incorporated procedural posture, procedure time, and operator experience as fixed effects and revealed that the seated posture was independently associated with smaller increases in both fatigue and pain compared with the standing posture.

For fatigue, seated posture was independently associated with reduced ΔFatigue (estimate −1.650, 97.5% CI −2.264 to −1.036, *p* < 0.0001). Procedure time was independently associated with greater increases in fatigue (estimate 0.012 per minute, 97.5% CI 0.001 to 0.024, *p* = 0.015), whereas operator experience was not significantly associated.

For pain, seated posture was similarly independently associated with reduced ΔPain (estimate −1.614, 97.5% CI −2.257 to −0.970, *p* < 0.0001). In contrast to fatigue, procedure time was not significantly associated with changes in pain ( *p* = 0.690), nor was operator experience ( Table 5 ). Additional mixed-effects linear regression analyses including an interaction term between procedural posture and procedure time showed no significant interaction for either ΔFatigue or ΔPain ( **Table S1** ).

**Table 5:** Linear mixed-effects model including an operator ID as a random intercept.

Variables	Estimate (ΔFatigue)	97.5% CI	*p* -Value	Estimate (ΔPain)	97.5% CI	*p* -Value
Posture (seated vs standing)	−1.650	−2.264 to −1.036	<0.0001	−1.614	−2.257 to −0.970	<0.0001
Procedure time (min)	0.012	0.001–0.024	0.015	0.002	−0.010 to 0.014	0.690
Operator experience (specialist vs. trainee)	0.219	−1.545 to 1.982	0.691	−0.048	−4.484 to 4.387	0.914

Δ values represent postprocedure minus preprocedure scores. CI, confidence interval.

In the sensitivity analysis restricted to the five operators who performed both seated and standing ERCP ( *n* = 100 procedures), the former was significantly associated with smaller increases in ΔFatigue and ΔPain, with comparable effect estimates as those of the primary analysis ( **Table S2** ).

### Secondary Outcomes

New-onset lower-limb discomfort occurred less frequently during seated ERCP than during standing ERCP, whereas no notable posture-related differences in the upper extremities or cervical symptoms were observed ( Table 6 ).

**Table 6:** Incidence of new-onset musculoskeletal symptoms by Anatomic region.

Region	Fatigue (seated), *n* (%)	Fatigue (standing), *n* (%)	*p* -Value	Pain (seated), *n* (%)	Pain (standing), *n* (%)	*p* -Value
Neck/Shoulder	3 (5.6%)	2 (4.4%)	0.669	2 (3.7%)	3 (5.1%)	1.000
Lower back	3 (5.6%)	3 (5.1%)	1.000	3 (5.6%)	4 (6.8%)	1.000
Lower limbs	0 (0%)	5 (8.5%)	0.058	0 (0%)	3 (5.1%)	1.000
Upper limbs	1 (1.9%)	0 (0%)	0.478	1 (1.9%)	0 (0%)	0.478

Operator-reported comfort was significantly higher during seated ERCP. The median comfort score was 5 (IQR, 4–5) for seated procedures and 3 (IQR, 2–3) for standing ones ( *p* < 0.0001), indicating that the former was perceived as substantially more comfortable.

## Discussion

In this single-center observational study, which was conducted under routine clinical practice conditions, seated ERCP was associated with smaller increases in ΔFatigue and ΔPain compared with the conventional standing posture without any apparent effect on procedural performance. This association was consistent across linear mixed-effects models analyzing operator-level variability. Thus, the ergonomic benefit of the seated posture could not be explained by individual operator factors. The association remained significant after adjusting for operator experience and procedure time, further substantiating the finding that procedural posture independently reduces operator burden during ERCP.

Notably, our findings indicate that fatigue and pain are influenced by partially distinct mechanisms. Adjusted analyses revealed an independent association between changes in fatigue and both procedural posture and time, whereas changes in pain were linked to posture alone. These findings imply that fatigue is influenced by cumulative physical workload accrued over the course of a procedure, whereas pain is more directly related to the sustained physiologic demands of operating posture during ERCP. Thus, beyond the intuitive relationship between longer procedures and greater fatigue, the ergonomics of the procedure appears to be a core factor in operator pain development, independent of procedural duration.

Several mechanisms can potentially explain the ergonomic advantage of seated ERCP. The requirement to wear heavy lead aprons under fluoroscopic guidance substantially increases axial loading and musculoskeletal strain, particularly during prolonged procedures. By transferring the weight of the protective apron from the operator’s spine to the operating chair, the seated posture reduces sustained axial loading and mechanical stress on the lumbar spine and lower extremities, thereby limiting fatigue accumulation during prolonged standing. These biomechanical considerations suggest that fatigue may contribute more significantly to cumulative axial load over time, whereas pain may be more directly related to sustained postural demands, independent of procedural duration. This procedure-specific working environment may partly explain the pronounced ergonomic effect of posture modification observed in our study.

Furthermore, all seated ERCP procedures were implemented using rotatable chairs as part of the procedural setup, facilitating operator positioning when shifting visual attention between the endoscopic and fluoroscopic monitors. Nonetheless, the current study did not evaluate the independent ergonomic contribution of chair mechanics. In contrast, standing ERCP requires additional postural adjustments. Because fluoroscopy room layout and monitor configuration vary across institutions, these considerations should be interpreted cautiously. Nevertheless, the findings highlight how the interaction between procedural environment and operator posture can affect the physical workload during ERCP.

The marked difference in operator-reported comfort scores further demonstrates the clinical relevance of posture modification. The higher comfort ratings observed during seated ERCP indicate that operators perceived the seated and standing postures as qualitatively distinct working conditions rather than minor ergonomic variations. Although a distinct, clinically relevant difference in operator fatigue was not established, the perceived reduction in fatigue and pain indicates a potentially relevant improvement in perceived operator comfort during routine clinical practice.

From a practical standpoint, seated ERCP is easier on the operator. Unlike interventions requiring specialized ergonomic devices or major workflow redesigns, posture modification in seated ERCP can be introduced at minimal cost and without altering the procedural technique. 17 18 Importantly, despite decreases in fatigue and pain levels, no differences were observed in procedure time, fluoroscopy-related parameters, or technical success. These findings address concerns that a seated posture might impair scope manipulation or situational awareness and support its selective adoption based on procedural complexity and physical demands.

The implications extend beyond short-term symptom relief. As ERCP operators age and procedural volumes increase, strategies that lower cumulative physical burden while maintaining procedural quality are becoming increasingly valuable for workforce sustainability. As musculoskeletal symptoms often emerge early in training, introducing ergonomically favorable postures during fellowship can help preserve long-term operator health and career longevity. 19 20 21 Although the present study focused on acute and procedure-related symptom changes rather than long-term musculoskeletal outcomes, the consistent association between a seated posture and reduced fatigue and pain supports the significance of posture modification within broader ergonomic practice in endoscopy units. Despite its potential ergonomic advantages, seated ERCP may not be widely adopted because of fluoroscopy room constraints (e.g., fixed equipment layout and limited space), long-standing operator habits favoring standing endoscopy, and perceived concerns about technical feasibility.

Several limitations warrant special consideration. First, this study had a single-center design and involved a limited number of operators, which can restrict generalizability. Second, fatigue and pain were assessed using a global NRS rather than region-specific or objective biomechanical measures, thereby limiting the accuracy of these evaluations. Future studies should incorporate objective physiologic metrics to quantify ergonomic effects more precisely. In addition, fatigue and pain were self-reported, and the operating posture could not be blinded. Therefore, reporting bias cannot be completely excluded. Third, although the values for fluoroscopy-related parameters were comparable between postures, actual operator radiation exposure and physical workload were not directly measured. 22 23 Fourth, fatigue and pain were assessed only immediately after ERCP and therefore reflected only acute changes. Future studies should evaluate symptom persistence and recovery over time using repeated assessments and objective ergonomic measures. Psychological factors, such as perceived stability or comfort associated with the seated posture, may also have influenced symptom reporting. Finally, the posture selection was determined by procedural circumstances and a room setup under routine clinical conditions rather than by randomization. Furthermore, ergonomic questionnaires were collected only when the investigator was available to administer the assessment. Therefore, not all eligible ERCP procedures performed during the study period were included, and selection bias cannot be completely excluded. Although baseline procedural characteristics were similar across the groups, the causal inference could not be definitively established due to the observational design. Nevertheless, paired pre- and postprocedure assessments alongside multivariate mixed-effects modeling—which accounted for operator-level variability—strengthen internal validity while reflecting real-world clinical practice.

In conclusion, seated ERCP was independently associated with reduced operator fatigue and pain levels, compared with the standing procedure, without compromising procedural performance. These findings support a seated posture as a practical and ergonomically advantageous technique in ERCP, while providing a foundation for future prospective and multicenter studies to further evaluate objective ergonomic metrics and long-term outcomes.

## Declaration of GenAI Use

The authors confirm that no Generative AI was used in the preparation of this manuscript.

## Supplementary Material

Ergänzendes Material Supplementary Material
