# Supplementary material for: Seated Versus Standing Endoscopic Retrograde Cholangiopancreatography: Association with Operator Fatigue and Pain
Source: Endosc Int Open. 2026 Sep 25;14:a29570590. [Article in German] doi: 10.1055/a-2957-0590 (PMC13643728; doi:10.1055/a-2957-0590)

Table S1. Mixed-effects linear regression analysis of changes in operator fatigue and pain after ERCP, including operator ID as a random intercept.

Variables	Estimate (ΔFatigue)	97.5% CI	p-value	Estimate (ΔPain)	97.5% CI	p-value
Posture (seated vs standing)	−1.638	−2.253 to −1.024	<0.0001	−1.611	−2.263 to −0.959	<0.0001
Procedure time (min)	0.018	0.001–0.036	0.020	0.004	−0.012 to 0.022	0.584
Operator experience (specialist vs. trainee)	0.207	−1.516 to 1.930	0.704	−0.057	−5.528 to 5.414	0.900
Posture × Procedure time	−0.010	−0.032 to 0.012	0.315	−0.004	−0.027 to 0.020	0.7123

Δ values represent post-procedure minus pre-procedure scores. CI: confidence interval

Endosc Int Open | DOI 10.1055/a-2957-0590 | © The Author(s).

Table S2. Sensitivity Analysis Incorporating a Linear Mixed-Effects Model (Restricted to Operators Who Performed Both Seated and Standing ERCP)

Variables	Estimate (ΔFatigue)	97.5% CI	p-value	Estimate (ΔPain)	97.5% CI	p-value
Posture (seated vs. standing)	−1.680	−2.336 to −1.023	<0.0001	−1.596	−2.295 to −0.898	<0.0001
Procedure time (min)	0.011	0.001 to 0.024	0.038	0.001	−0.012 to 0.014	0.892
Operator experience (specialist vs. trainee)	0.605	−1.986 to 3.195	0.389	0.149	−9.831 to 10.128	0.769

Δ values represent post-procedure minus pre-procedure scores. ERCP: endoscopic retrograde cholangiopancreatography; CI: confidence interval

Figure S1.

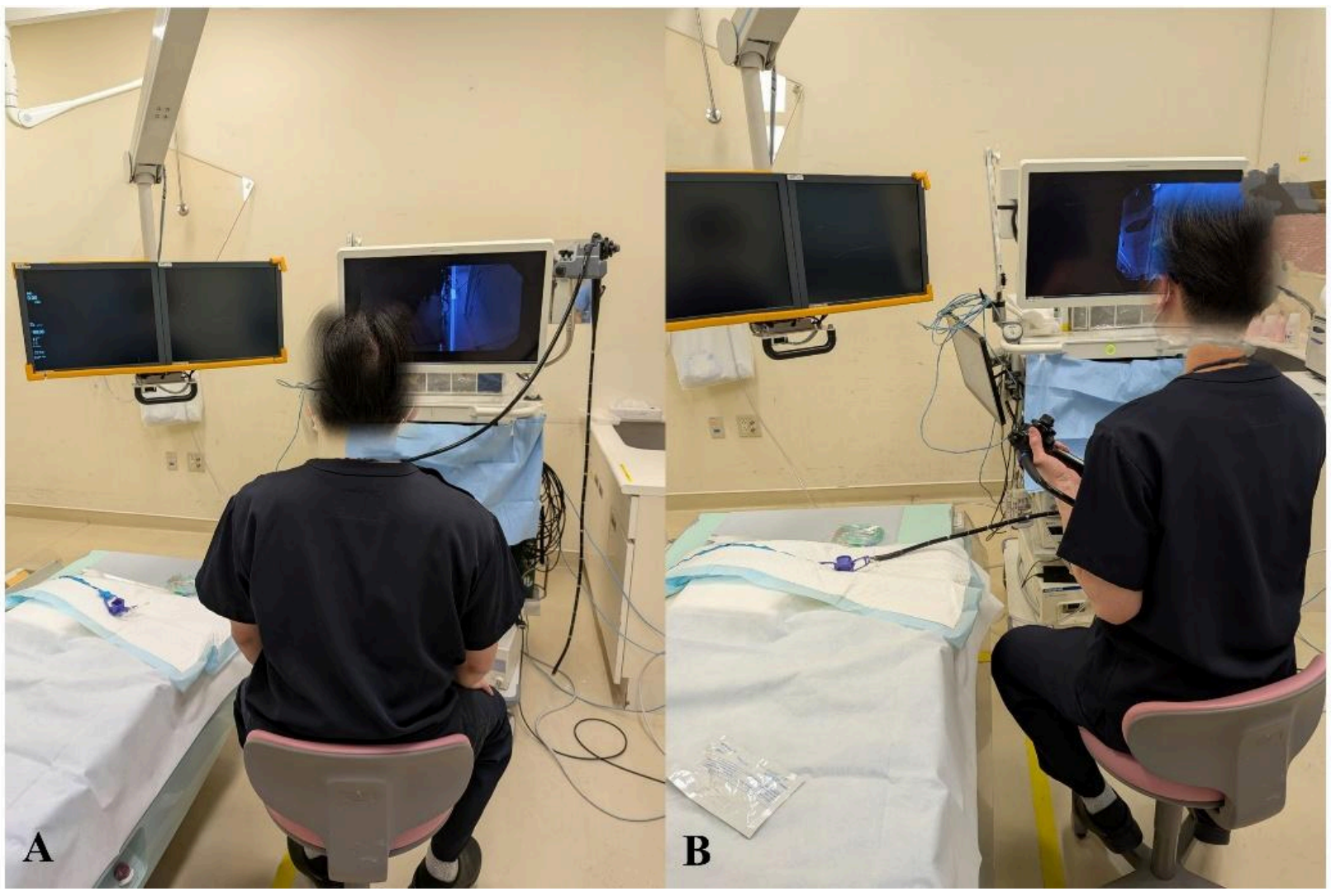

Figure S2.

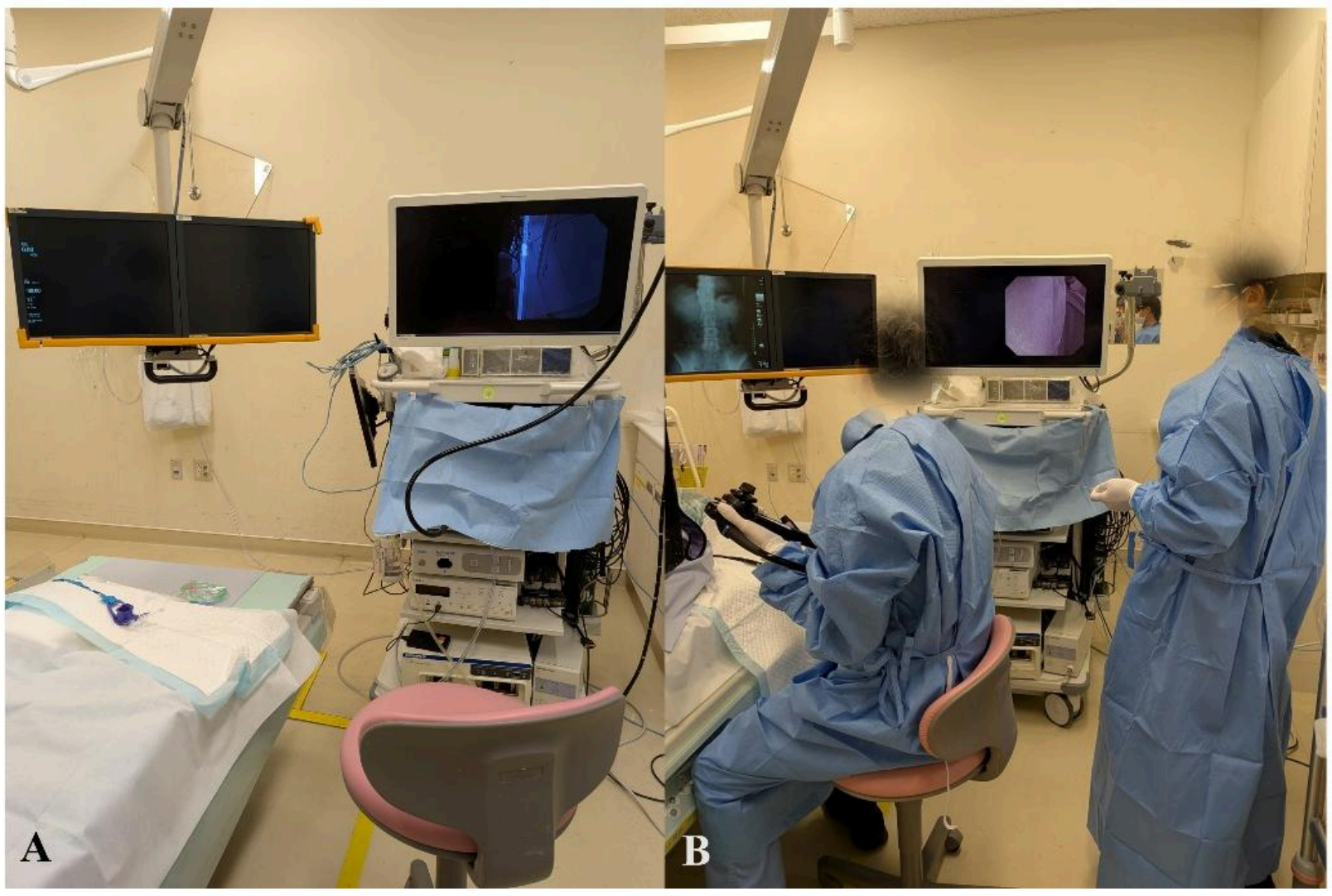

Supplement: Supplementary file 1 [file 10-1055-a-2957-0590_29574974.pdf]
